# The Psychopathology in Autism Checklist–Revised (PAC-R): Development and Validation

**DOI:** 10.1177/13623613261480174

**Published:** 2026-08-30

**Authors:** Arvid Nikolai Kildahl, Sissel Berge Helverschou, Børge Mathiassen, Einar Heiervang, Marianne Berg Halvorsen

**Affiliations:** 1 Oslo University Hospital, Asker, Norway; 2 University Hospital of North Norway, Tromsø, Norway; 3 UiT The Arctic University of Norway, Tromsø, Norway; 4 Innlandet Hospital Trust, Tynset, Norway; 5 Nordland Hospital Trust, Bodø, Norway; 6 Finnmark Hospital Trust, Hammerfest, Norway; 7 Helgeland Hospital Trust, Mo i Rana, Norway

**Keywords:** autism, intellectual disability, mental health disorder, screening, assessment

## Abstract

There are few appropriate screening tools for mental health in autistic people, particularly for individuals with co-occurring intellectual disabilities. A specially developed screening tool for this population, the Psychopathology in Autism Checklist (PAC), has shown acceptable psychometric properties. However, a recent study showed that the subscale structure of the PAC was not in line with its underlying factor structure. The current study aimed to examine the screening properties of a restructuring of the PAC items in line with its factor structure, resulting in a revised version of the PAC, the PAC–Revised (PAC-R). Cut-offs for the PAC-R subscales were identified in a high-risk sample of autistic adults and older adolescents with intellectual disabilities who had been referred for mental health assessment (*n* = 182; Sample 1). The psychometric properties of these cut-offs and the statistical fit of the subscales were investigated in this sample, as well as in a low-risk sample of autistic children and adolescents (*n* = 197; Sample 2). This revised version of the PAC, referred to as the PAC-R and comprising five subscales (obsessive-compulsive disorder, psychosis, anxiety, depression, aggression/self-injurious behaviours) and an “any mental health disorder” index, appeared to retain or improve on the screening properties of the original PAC in Sample 1. Similar findings were made for the anxiety and depression subscales and the “any mental health disorder” index in Sample 2. Statistical fit of the model was acceptable across both samples. These findings indicate that the PAC-R is an applicable mental health screening tool for autistic adults with intellectual disabilities, as well as for anxiety and depression in autistic children and adolescents with varied intellectual functioning.

**Lay Abstract:**

Despite autistic people being at increased risk of co-occurring mental health disorders, there are few assessment tools developed specifically for the identification of mental health disorders in autistic people. This lack of available tools is especially problematic for autistic individuals with co-occurring intellectual disabilities, who may have difficulties answering questions about potential mental health symptoms, or may display symptoms of mental health disorder in atypical ways. The Psychopathology in Autism Checklist (PAC) has previously been rated as a promising tool for this population. However, a recent finding suggested that the items of the PAC do not seem to be grouping in the way it was presumed when the PAC was first developed. The current study describes the revision of the PAC into the PAC–Revised (PAC-R) and its validation across two different samples. These samples included a high-risk sample for mental health disorder, autistic adults and older adolescents with co-occurring intellectual disabilities referred for mental health assessment, and a lower-risk sample for mental health disorder, autistic children and adolescents referred for initial autism diagnostic assessment. Across both samples, the PAC-R appeared to retain or improve on the properties of the original PAC as a screening tool. In addition, the subscale structure of the PAC-R provided an improved fit to the data with regard to the grouping of the items. In conclusion, the PAC-R appears to be a promising measure for screening for mental health disorders in autistic individuals. However, comprehensive and multimethod assessments remain necessary for making an accurate mental health diagnosis, as the PAC-R is intended for screening purposes only.

## Introduction

The prevalence of co-occurring mental health disorders among autistic people is high (Hollocks et al., 2019; Lai et al., 2019; Rosen et al., 2018), with up to 70% of autistic individuals meeting criteria for at least one such disorder and almost 50% for more than one (Lai et al., 2019). However, assessment of mental health in this population may be challenging (Helverschou et al., 2011; Lai et al., 2019), in part, due to a lack of appropriate assessment tools (Halvorsen, Kildahl, Kaiser, et al., 2025). This lack of assessment tools includes both screening tools and more comprehensive diagnostic assessment tools and appears to be particularly pronounced for individuals with co-occurring intellectual disabilities (Halvorsen, Kildahl, Kaiser, et al., 2025).

Lai et al. (2019) have highlighted that the high prevalence of mental health disorders among autistic people “implies that mental health assessment should be an integral aspect of clinical care with regular screening, evaluation, and treatment” (p. 826). However, regular screening requires screening tools that have been validated in autistic individuals. The use of tools that have not been developed for or adapted to autistic individuals may involve a risk of misdiagnosis (Halvorsen, Kildahl, Kaiser, et al., 2025), including diagnostic overshadowing (Jopp & Keys, 2001; Reiss et al., 1982), wherein symptoms of a co-occurring mental health disorder are misinterpreted and misattributed to the individual’s autism or their co-occurring intellectual disability, or overdiagnosis if the individual’s autism characteristics are not sufficiently accounted for during assessment (Helverschou et al., 2011).

One of the very few mental health screening tools developed specifically for autistic people from the outset is the Psychopathology in Autism Checklist (PAC), which was originally developed for autistic adults with intellectual disabilities (Helverschou et al., 2009; Helverschou, Ludvigsen et al., 2021). A recent review (Halvorsen, Kildahl, Kaiser, et al., 2025) identified the PAC as a promising measure in terms of preliminary evidence of its reliability and validity, while highlighting that more studies are needed to evaluate its psychometric properties. The PAC was constructed by employing a theoretical and nosological approach, using expert judgement to identify symptoms of four specific mental health disorders that had minimal overlap with autism characteristics (Halvorsen, Kildahl, Kaiser, et al., 2025; Helverschou et al., 2008, 2009). It comprises 42 items, 30 of which are distributed across four disorder-specific subscales: psychosis (10 items), depression (7 items), anxiety (6 items), and obsessive-compulsive disorder (OCD, 7 items). In addition, symptoms that were rated by experts as potential indicators of more than one of these disorders were included in a general adjustment problems (GAP) subscale comprising 12 items, including irritability, sleep disturbance, and self-injurious behaviour.

The 42 PAC items are rated in two domains: “Extent of problems” is rated on a 4-point Likert-type scale (“not a problem” = 1, “minor problem” = 2, “moderate problem” = 3, “severe problem” = 4), while “change from usual behaviour” is rated on a 3-point Likert-type scale (worsened, unchanged, improved). Scoring involves calculating the mean of the “extent of problems” scores for each subscale, and these mean scores are used in a two-step procedure to identify individuals considered screen positive for a mental health disorder. First, it is considered whether the individual has a GAP score above the identified cut-off value for this subscale, and then whether the individual also has a score above the respective cut-off values for any of the four disorder-specific subscales (Helverschou et al., 2009).

The cut-off values for the PAC were identified by Helverschou et al. (2009) in a sample of autistic adults with intellectual disabilities who had been diagnosed with the four disorders in question. A later study (Helverschou, Ludvigsen et al., 2021) found these cut-off values to be helpful in screening for all mental health disorders in autistic adults and older adolescents with intellectual disabilities, concluding that the PAC had potential value as a screening tool, while also highlighting that clinical diagnoses should be based on multimodal and multidisciplinary assessments. Notably, the PAC appeared to have slightly stronger discriminative properties among individuals with severe/profound intellectual disabilities, compared to those with mild/moderate intellectual disabilities. While the PAC was originally developed for autistic adults with intellectual disabilities, recent findings suggest that it may also be applicable in autistic children and adolescents (Halvorsen, Kildahl, Thimm, et al., 2025), including those without co-occurring intellectual disabilities and intellectual functioning in the low average to average range.

Despite these promising findings with regard to its properties as a screening tool, a recent study of the factor structure of the PAC (Kildahl et al., 2026) showed that the GAP subscale does not appear to work as intended. Rather than loading on multiple subscales, the GAP items appeared to comprise a mix of items loading with only one of the other subscales and items loading on a separate aggression and self-injurious behaviour factor. Moreover, the GAP subscale did not appear to constitute a cohesive concept and showed poor fit with the data. In addition, two of the subscales (psychosis, anxiety) did not show longitudinal measurement invariance, meaning that they do not appear to be appropriate for use in the evaluation of treatment and intervention in their current form. However, the results from an exploratory factor analysis intended to investigate the distribution of the GAP subscale items resulted in an alternative grouping of the items (Kildahl et al., 2026), a grouping in which all five subscales showed longitudinal measurement invariance. This involved retaining the same four disorder-specific subscales, though with slightly different item configurations. While these findings may give cause for optimism, it is necessary to investigate whether this alternative grouping of the items is also appropriate for screening purposes.

In this study, we aim to develop a revised version of the PAC, the PAC-R. If found to be suitable for mental health screening, this new revision of the PAC will result in a psychometrically sound instrument suitable for screening as well as for evaluation of intervention, which the original PAC appears not to be. To achieve this, this first study of the PAC-R will examine whether the alternative subscale structure proposed by Kildahl et al. (2026) may be suitable for screening purposes across two independent samples. Sample 1 is a high-risk sample for mental health disorders and is the sample in which the alternative subscale structure was first identified: a sample of autistic individuals with intellectual disabilities referred for mental health assessment. Sample 2 is a sample of autistic children and adolescents who were screened using the PAC after being referred for autism assessment, that is, a sample with presumably lower risk of mental health disorders.

The research questions were as follows:

**Research Question 1:** What are the optimal cut-off values for the PAC-R according to clinical diagnoses in Sample 1?
**Research Question 2:** Is the PAC-R as precise as the original PAC in screening for mental health disorders in Sample 1?
**Research Question 3:** What is the statistical fit of the PAC-R in Sample 2?
**Research Question 4:** What are the psychometric properties of the PAC-R in Sample 2, including the cut-off values identified in Sample 1?

## Methods

### Design/Setting

#### Sample 1: High-Risk Adult Sample

Sample 1 was obtained from the AUP (autism, intellectual disability, mental illness) multicentre study (Helverschou, Bakken et al., 2021), a longitudinal and descriptive study of mental health assessment and treatment of autistic adults and older adolescents with intellectual disabilities. Eight centres throughout Norway collected data for individuals in this population referred for mental health assessment, and/or due to challenging behaviour. Participants in this sample were referred for assessment/treatment of a possible co-occurring mental health disorder, meaning the sample constitutes a high-risk sample for mental health problems. Data were collected across three time points (intake, post-intervention/discharge, follow-up 12 months after discharge) between 2010 and 2020. For the current study, PAC scores from T1 were used alongside the clinical diagnoses reported at T2. For further details concerning the AUP multicentre study, see Helverschou, Bakken et al. (2021).

#### Sample 2: Low-Risk Child/Adolescent Sample

Sample 2 was obtained from a study on autism and mental health in children and adolescents (Halvorsen, Heiervang, et al., 2025; Halvorsen, Kildahl, Thimm, et al., 2025), involving all public health trusts conducting autism assessments in children and adolescents in Northern Norway. Participants in this sample were referred for autism assessment, but not because of suspected co-occurring mental health difficulties, meaning the sample presumably constitutes a low-risk sample for mental health difficulties. Data collection was conducted between 2020 and 2024 at one of the locations and between 2022 and 2024 at the remaining three locations. For further details concerning the study, see Halvorsen, Heiervang, et al. (2025) and Halvorsen, Kildahl, Thimm, et al. (2025).

### Participants

#### Sample 1: High-Risk Adult Sample

All participants in the AUP study data set with completed T1 PACs and T2 clinical diagnosis reports were included in the current study, resulting in a total sample of 182 (56 females, 126 males). Ages ranged from 13 to 68 years (*M* = 27.60, *SD* = 10.58); 118 participants had mild or moderate intellectual disabilities, while 64 participants had severe or profound intellectual disabilities. The prevalence of co-occurring mental health disorder was high in this sample, with 116 (63.7%) participants diagnosed with at least one mental health disorder. This included all mental health disorders, but not attention-deficit/hyperactivity disorder (ADHD), which is defined as a neurodevelopmental disorder.

#### Sample 2: Low-Risk Child/Adolescent Sample

All participants diagnosed with autism and who completed PACs were included in the current study, resulting in a total sample of 197 (57 females, 140 males). Ages ranged from 2 to 19 years (*M* = 8.36, *SD* = 4.74). Forty-one (21.7%) participants had been diagnosed with a co-occurring intellectual disability (27 mild, 2 moderate, 4 severe, 8 unspecified), while 156 participants had not. For the purpose of the current study, oppositional defiant disorder (ODD) and ADHD were disregarded as mental health diagnoses, as the PAC-R is not developed to identify co-occurring neurodevelopmental or conduct disorders in autistic individuals. Therefore, according to the results from the Developmental and Well-Being Assessment (DAWBA; Goodman et al., 2000), the number of participants meeting the criteria for at least one co-occurring mental health disorder in this sample was 48 (27.7%). This included all mental health disorders other than ADHD and ODD.

### Research Ethics

#### Sample 1

The AUP multicentre study, from which the data for Sample 1 for the current study were obtained, was approved by the Data Protection Officer, Oslo University Hospital, Oslo, Norway, #2010/19579. Due to most participants having limited capacity to consent, written informed consent was obtained from their legal guardians. Participants who were able to also gave their own written consent. Potential adult participants who were able to consent but did not wish to participate were not included, irrespective of whether their legal guardians had consented.

#### Sample 2

The study providing the data for Sample 2 in the current study was approved by the Regional Committee for Medical and Health Research Ethics in North Norway, #41098, as well as the data protection officers at all participating hospitals. Written informed consent was obtained from the parents of the participants. In addition, participants aged 16 years and older gave their own consent.

### Materials

#### PAC-R

The original PAC is a mental health screening checklist originally developed for autistic adults with intellectual disabilities (Helverschou et al., 2008, 2009). While regarded as a promising measure (Halvorsen, Kildahl, Kaiser, et al., 2025), several aspects of its psychometric properties are yet to be investigated. Each item on the 42-item checklist is rated in two domains: “extent of problems” and “change from usual behaviour.” “Extent of problems” is scored on a 4-point Likert-type scale from 1 (*not a problem*) to 4 (*severe problem*). Mean scores for each of the five subscales (GAP, psychosis, depression, anxiety, OCD) are calculated. A score is considered screen positive only if the individual’s mean scores on both the GAP and one of the four disorder-specific subscales are above the specified cut-off values (Helverschou et al., 2009).

The PAC-R is a further development of the PAC, wherein items have been restructured into revised subscales according to findings regarding their underlying factor structure, as shown by an exploratory factor analysis (Kildahl et al., 2026). The two-step procedure of the original PAC involving the GAP subscale has been discontinued, and the PAC-R has five potential subscales (psychosis, depression, anxiety, OCD, aggression/self-injurious behaviours), and the composition of these subscales differs from those of the original PAC (see Table 1). The PAC GAP subscale (12 items) has been abolished, but four of these items are included in the PAC-R aggression/self-injurious behaviours subscale (6 items), along with two items from the PAC psychosis scale. Four items have been removed from the PAC psychosis subscale (10 items) to form the PAC-R psychosis subscale (6 items). The PAC OCD subscale (7 items) has retained five of the same items in the PAC-R OCD subscale, with the addition of one item from the PAC GAP subscale. Two items from the PAC GAP subscale were moved to the PAC-R anxiety subscale (9 items), and two were moved to the PAC-R depression subscale (8 items). In addition, two items originally placed in the PAC psychosis subscale were shown to load with the anxiety items, resulting in both the PAC-R depression and anxiety subscales comprising more items than their original counterparts, the PAC depression (7 items) and PAC anxiety (6 items).

**Table 1.** Item Composition by Psychopathology in Autism Checklist–Revised Subscale.

Subscale	Item short form (original subscale)
Aggression/self-injurious behaviours	6. Reacts with irritation (GAP) 7. Self-injures (GAP) 9. Destroys surroundings (GAP) 11. Violent towards other people (GAP) 23. Acts in a threatening manner (Psychosis) 26. Has sudden changes of mood (Psychosis)
OCD	14. Is excessively concerned (OCD) 19. Checks repeatedly (OCD) 22. Has difficulties stopping (OCD) 27. Has a special behaviour pattern (OCD) 40. Needs to carry . . . (OCD) 42. Spends more time . . . (GAP)
Psychosis	8. Loses the thread or track (Psychosis) 13. Sees things . . ./visual hallucinations (Psychosis) 17. Gets disoriented in familiar . . . (Psychosis) 29. Auditory hallucinations (Psychosis) 34. Is disoriented/confused . . . (Psychosis) 37. Is unable to follow familiar . . . (Depression)
Depression	1. Is passive (GAP) 2. Takes little initiative (GAP) 12. Appears to be tired (Depression) 20. Needs a great deal of help (Depression) 24. Shows loss of energy (Depression) 30. Has gained/lost weight (Depression) 35. Shows little interest in . . . (Depression) 39. Appears sad and dejected (Depression)
Anxiety	3. Appears agitated (GAP) 10. Is abnormally anxious . . . (GAP) 15. Shakes/trembles (Anxiety) 18. Hyperventilates (Anxiety) 25. Sweats, feels clammy (Anxiety) 28. Shows fear of specific situations (Anxiety) 32. Appears nervous (Anxiety) 38. Has a high level of motor activity (Psychosis) 41. Has a high level of communication (Psychosis)
Any mental health disorder	All items from the OCD, Psychosis, Depressive disorder, and Anxiety disorder subscales, plus: 4. Has difficulty sleeping (GAP) 5. Withdraws from social situations (GAP) 16. Complains about pains (GAP)

*Note.* Obsessive-compulsive disorder (OCD) and general adjustment problems (GAP). The original subscale placement of each item is presented in parentheses. Four items of the original PAC were not included in the PAC-R due to low factor loadings or conceptual inconsistencies; see Kildahl et al., (2026): 21. Delusions (Psychosis), 31. Has enlarged pupils (Anxiety), 33. Repeatedly washes things (OCD), and 36. Excessive washing (OCD).

#### Mental Health Diagnoses (Sample 1)

The mental health diagnoses in Sample 1 were clinical diagnoses based on the results of comprehensive assessments, including the AUP multicentre study protocol, and also included the use of clinical observation and additional assessment tools (Helverschou, Bakken, et al., 2021; Helverschou, Ludvigsen et al., 2021). In other words, reported diagnoses were made following standard clinical practice, and the teams conducting the comprehensive assessments were not blind to the PAC scores of the respective participants. Diagnoses were reported by the clinicians in charge of the respective assessment (psychiatrist/clinical psychologist) at T2.

#### Mental Health Diagnoses (Sample 2)

Mental health diagnoses in Sample 2 were derived from the DAWBA (Goodman et al., 2000). Three different versions of the DAWBA were used: a parent version completed by all parents, a youth version for children aged 11 and older, and a teacher version. The Norwegian-language version has demonstrated realistic prevalence estimates for mental health disorders, as well as high predictive validity when used in public health services (Brøndbo et al., 2012; Heiervang et al., 2007). Two research-reliable clinicians (BM, ERH), both blind to PAC scores, generated diagnostic ratings based on a combination of responses provided by youths (*n* = 42), parents (*n* = 174), and teachers (*n* = 137) to structured (yes/no) and open-ended questions. Diagnostic decisions were guided by the *Diagnostic and Statistical Manual of Mental Disorders* (4th ed.; DSM-IV criteria; American Psychiatric Association, 1994), with comorbidity recorded when the criteria for more than one diagnosis were met, disregarding the exclusion rules of the DSM-IV. Completed DAWBAs were missing for 23 participants. The inter-rater reliability for DAWBA diagnoses between BM and ERH in similar samples has been reported as ranging from adequate to excellent (Brøndbo et al., 2012). Oppositional defiant disorder and ADHD were disregarded as mental health diagnoses, as the PAC-R is not developed to identify co-occurring neurodevelopmental or conduct disorders in autistic individuals.

### Procedure

Data for both samples were obtained during regular clinical practice (see Helverschou, Bakken et al., 2021; Halvorsen, Heiervang, et al., 2025). For Sample 2, study data were managed using Research Electronic Data Capture (REDCap) tools hosted at the University Hospital of North Norway (Harris et al., 2009).

### Analysis

The statistical analyses were conducted in SPSS 30, except for the analyses of model fit (confirmatory factor analysis [CFA]), which were conducted in R. In Sample 1, missing PAC items were managed as described in a previous study (Kildahl et al., 2026), by replacing the missing value with the subscale mean rounded up or down to the closest whole integer. For the original PAC subscales, 24 item responses (0.31%) were imputed in this manner, while 14 item responses (0.20%) were imputed for the PAC-R subscales. The discrepancy was caused by two of the four PAC items that were not included in the PAC-R, accounting for a substantial proportion of missing responses. In Sample 2, the number of imputations was limited to a few items on a total of two PAC forms.

To identify the optimal cut-off values of the PAC-R subscales, the means of the anxiety, psychosis, depression, and OCD subscales were calculated for participants in Sample 1 who were diagnosed with the respective disorder and those who were not. For example, the mean scores on the psychosis subscale in the participants diagnosed with psychotic disorders (*N* = 31) and the remaining sample (*N* = 151) were 2.25 and 1.77, respectively. Then, the sensitivity, specificity, positive predictive value (PPV), negative predictive value (NPV), and area under the curve (AUC) were calculated for potential cut-off values between these two means for each subscale. For the psychosis scale, examples of these calculations can be seen in Table 2. In the choice of cut-off value, the breadth of the information from these analyses was considered, while aiming to achieve the best possible balance between sensitivity and specificity while slightly emphasising sensitivity, as the PAC is primarily intended for use as a screening tool. No cut-off value was calculated for the aggression/self-injurious behaviour subscale, because its scores did not significantly differ between the groups. In addition, sensitivity, specificity, PPV, NPV, and AUC were calculated for the original subscales to compare these with the results of using the PAC-R for screening. Because the participants diagnosed with psychotic disorders had very high scores on the PAC anxiety subscale (Kildahl et al., 2026), additional exploratory analyses were conducted in which these participants were included alongside those diagnosed with anxiety disorders for the anxiety subscales. In addition, the psychometric properties of the cut-off values were examined in separate analyses for individuals with mild/moderate and severe/profound intellectual disabilities, respectively.

**Table 2.** Psychometric Properties Used in Determination of the Cut-off for the PAC-R Psychosis Scale.

Cut-off	Sensitivity	Specificity	PPV	NPV	AUC
1.8	.65	.60	.25	.89	.621
1.9	.52	.66	.24	.87	.589
2.0	.52	.66	.24	.87	.589
2.1	.48	.72	.26	.87	.600

*Note.* A cut-off of 1.8 was chosen for this subscale due to the sensitivity and the AUC being higher for this cut-off than the other choices.

As for screening for “any mental health disorder,” the original version of the PAC treats this as equal to being screen positive for one of the more specific disorders. However, because there are only four subscales reflecting more specific disorders, and there are other relevant mental health diagnoses that may not be captured by these subscales, we wanted to use a more overarching strategy for general screening. We therefore chose to calculate the sum of items on the four disorder-specific PAC-R subscales, in addition to three items from the original PAC that did not load on any of the PAC-R subscales but were assumed to be non-specific signs of discomfort (sleep difficulties, complaints about pain, social withdrawal). We then identified potential cut-off values for the sum of all item scores in the same manner as for the individual subscales. The identified cut-off value was then compared to the “any mental health disorder” procedure of the original PAC.

To investigate model fit for the PAC-R in Sample 2, CFA was conducted for this model in this sample and compared to the fit indices in Sample 1. For further investigation of the psychometric properties of the PAC-R in Sample 2, McDonald’s omega (ω) was calculated for each subscale, in addition to sensitivity, specificity, PPV, NPV, and AUC for each subscale, and “any mental health disorder” in relation to the diagnoses derived from the DAWBA.

### Participatory Methods Statement

The study from which Sample 2 was obtained was conducted in collaboration with a representative of the Norwegian Autism Association (see Acknowledgements).

## Results

The optimal cut-off values identified for the PAC-R in Sample 1 were 1.8 for the psychosis subscale, 2.1 for the anxiety subscale, 2.2 for the depression subscale, and 2.3 for the OCD subscale. In addition, a cut-off value of 1.8 for “any mental health disorder” was identified. The psychometric properties of these cut-off values in Sample 1 are presented in Table 3, alongside the psychometric properties of the original PAC subscales in the same sample. When the participants diagnosed with a psychotic disorder were included with those diagnosed with an anxiety disorder in the calculations regarding the anxiety subscale, the PPV in particular appeared to improve. Overall, most of these values were similar to the original PAC subscales (see Table 3).

**Table 3.** Screening Properties of the PAC-R and the Original PAC Subscales in Sample 1.

		Alternative subscale structure (PAC-R)					Original subscales				
	Diagnosed (%)	Sensitivity	Specificity	PPV	NPV	AUC	Sensitivity	Specificity	PPV	NPV	AUC
Full sample (*n* = 182)											
Psychosis	31 (17.0%)	.65	.60	.25	.89	.621	.60	.74	.32	.90	.672
Depression	44 (24.2%)	.55	.49	.26	.77	.519	.58	.50	.28	.78	.541
Anxiety	45 (24.7%)	.71	.54	.34	.85	.626	.71	.63	.39	.89	.669
Anxiety’	75 (41.2%)	.69	.60	.55	.74	.646	.65	.68	.58	.74	.662
OCD	12 (6.6%)	.75	.68	.14	.97	.713	.50	.85	.19	.96	.674
Any mental health disorder	116 (63.7%)	.78	.47	.72	.54	.623	.75	.50	.73	.53	.625
Mild/moderate intellectual disabilities (*n* = 118)											
Psychosis	27 (22.9%)	.59	.65	.33	.84	.620	.58	.82	.47	.87	.696
Depression	31 (26.3%)	.58	.51	.30	.77	.543	.59	.54	.32	.78	.564
Anxiety	26 (22.0%)	.69	.52	.29	.86	.607	.62	.62	.31	.85	.617
Anxiety’	53 (44.9%)	.68	.60	.58	.70	.640	.58	.68	.59	.67	.629
OCD	8 (6.8%)	.75	.72	.16	.98	.734	.38	.86	.16	.95	.615
Any mental health disorder	79 (66.9%)	.75	.49	.75	.49	.617	.70	.51	.74	.45	.605
Severe/profound intellectual disabilities (*n* = 64)											
Psychosis	4 (6.3%)	1.00	.52	.12	1.00	.758	.75	.63	.12	.97	.692
Depression	13 (20.3%)	.46	.47	.18	.77	.466	.54	.45	.20	.79	.495
Anxiety	19 (29.7%)	.74	.58	.42	.84	.657	.84	.64	.50	.91	.743
Anxiety’	22 (34.4%)	.73	.60	.48	.81	.661	.82	.67	.56	.88	.742
OCD	4 (6.3%)	.75	.60	.11	.97	.675	.75	.83	.23	.98	.792
Any mental health disorder	37 (57.8%)	.84	.44	.67	.67	.641	.87	.48	.70	.72	.673

*Note.* Psychopathology in Autism Checklist (PAC), Psychopathology in Autism Checklist–Revised (PAC-R), and obsessive-compulsive disorder (OCD). Anxiety’ includes participants diagnosed with psychotic disorders in the target group, as most of these patients had elevated scores on the anxiety subscale.

CFA for the five-factor PAC-R subscale structure in Sample 2 resulted in acceptable fit indices, χ^2^(550, *N* = 196) = 928.28, *p* < .001 (root mean square error of approximation [RMSEA] = 0.059, RMSEA 90% confidence interval [CI] = [0.053, 0.066], Tucker–Lewis index [TLI] = 0.852, comparative fit index [CFI] = 0.863), improved from a one-factor solution, χ^2^(560, *N* = 196) = 1,097.28, *p* < .001 (RMSEA = 0.070, RMSEA 90% CI = [0.64, 0.076], TLI = 0.794, CFI = 0.806). Fit indices for the PAC-R five-factor solution in Sample 2 were similar to fit indices in Sample 1, χ^2^(550, *N* = 182) = 822.71, *p* < .001 (RMSEA = 0.052, RMSEA 90% CI = [0.045, 0.060], TLI = 0.893, CFI = 0.901).

The internal consistency of the PAC-R subscale structure across the two samples is presented in Table 4. The internal consistency of these subscales varied from good to excellent, with only two subscales showing internal consistency < .70 in one of the samples. Finally, the psychometric properties of the cut-off values identified in Sample 1 are presented for Sample 2 in Table 5, alongside the psychometric properties of the original PAC subscales.

**Table 4.** Internal Consistency (McDonald’s ω) of the PAC-R Subscales.

	Sample 1 (*n* = 182)	Sample 2 (*n* = 197)
Psychosis	.78	.64
Depression	.77	.80
Anxiety	.75	.68
OCD	.79	.73
Aggression/self-injurious behaviour	.82	.74
Any mental health disorder	.88	.88

*Note.* Psychopathology in Autism Checklist–Revised (PAC-R) and obsessive-compulsive disorder (OCD).

**Table 5.** Screening Properties of the PAC-R and the Original PAC Subscales in Sample 2 (*n* = 173).

		Alternative subscale structure (PAC-R)					Original subscales				
	Diagnosed (%)	Sensitivity	Specificity	PPV	NPV	AUC	Sensitivity	Specificity	PPV	NPV	AUC
Psychosis	0 (0.0%)										
Depression	8 (4.6%)	.88	.73	.13	.99	.801	.63	.73	.10	.98	.679
Anxiety	47 (27.2%)	.60	.81	.54	.84	.703	.49	.90	.64	.82	.693
OCD	2 (1.2%)	1.00	.89	.11	1.00	.947	1.00	.97	.25	1.00	.982
Any mental health disorder	48 (27.7%)	.69	.70	.46	.85	.692	.58	.77	.49	.83	.676

*Note.* Psychopathology in Autism Checklist (PAC), Psychopathology in Autism Checklist–Revised (PAC-R), and obsessive-compulsive disorder (OCD).

No participants in Sample 2 met the criteria for a psychotic disorder. Therefore, analyses of sensitivity/specificity were not conducted for this subscale in Sample 2. Moreover, only two participants met the criteria for OCD, meaning the results concerning this subscale should be interpreted with caution. While the accuracy of the anxiety subscale appeared to be similar between the PAC and the PAC-R, the accuracy of the PAC-R depression subscale was improved compared to the original PAC depression subscale.

## Discussion

The current study found that the PAC-R retains or improves on the PAC’s properties as a mental health screening tool in autistic individuals with intellectual disabilities (Sample 1), indicating that these empirically developed subscales are preferable to the original subscales. While originally developed for mental health screening in autistic adults with intellectual disabilities, the current results further indicate that the PAC-R is appropriate for use in autistic children and adolescents. Moreover, these findings show that the PAC-R is preferable to the original PAC when screening for anxiety and depressive disorders in autistic children and adolescents, while data on psychosis and OCD were lacking for this sample. In sum, these findings highlight the potential of the PAC-R as a screening tool for mental health disorders in autistic people. However, there seems to be a potential risk of false positives on the four disorder-specific subscales, as reflected by the less-than-optimal PPVs across both samples, and a risk of false negatives on the “any mental health disorder” score, as reflected by the low NPV in Sample 1. Therefore, these findings highlight the importance of conducting comprehensive and multimodal mental health assessments for individuals who are screen positive on the PAC-R. Scores on tools such as the PAC-R should be interpreted in the context of other information, and item-level analysis may be necessary to minimise the risk of overlooking cases of mental health disorder in autistic people in clinical practice.

The model fit of the five-subscale structure of the PAC-R was acceptable across both samples, though slightly poorer in the child/adolescent sample. This further highlights the potential applicability of the PAC-R in autistic children and adolescents with varied intellectual functioning, as previously shown for the PAC by Halvorsen, Kildahl, Thimm et al. (2025). Their findings further suggest that the slightly poorer fit indices of the PAC-R in the child/adolescent sample may be due to the fact that no participant in this sample met the criteria for a psychotic disorder. However, further studies investigating the psychometric properties of the PAC-R among autistic children and adolescents are needed. In addition to acceptable fit, the scoring procedure of the PAC-R has been simplified compared to the original PAC, eliminating the two-step procedure, and only involves calculating separate scores for the four disorder-specific subscales and an “any mental health disorder” score. These simplifications, and the removal of four items, should make the PAC-R easier and more time-efficient to use as a screening tool – as it now only requires 38 items to be scored on a 4-point Likert-type scale, with an optional section for rating whether the symptoms/behaviours have worsened, improved, or remained the same. The latter section was not used for the calculations in the current study, and further studies are needed to identify how clinicians may integrate scores from this section in their clinical decision-making.

While the PAC-R appeared to be applicable across both samples, there was some variation. For example, for the PAC-R anxiety subscale, the psychometric properties appeared to be similar, while these were far better for the depression subscale in Sample 2. It is possible that the subscale is more suited to capture symptoms of depression in autistic children and adolescents. However, this would be surprising, seeing as the subscale was originally developed for autistic adults with intellectual disabilities and was constructed to capture symptoms of depression in individuals with limited verbal language skills. The diagnostic process in Sample 2, using diagnoses derived from the DAWBA, was more structured and systematic than the clinical diagnoses reported in Sample 1, for which the clinicians in question were not blind to the PAC scores. Thus, depressive disorders may have been underdiagnosed in Sample 1, in line with previous accounts of potential underdiagnosis of mental health disorders in autistic individuals with intellectual disabilities (Kildahl et al., 2024; Peña-Salazar et al., 2022), and this may have contributed to the poorer psychometric properties of this subscale in Sample 1. However, it is not clear why this would only have affected the depression subscale, unless the risk of overlooking depression qualitatively differs from the risk of overlooking other mental health disorders. Such potential differences in the risk of diagnostic overshadowing could be due to potential overlap in behavioural manifestations of depression, lack of recognition of depressive symptoms in individuals with severe/profound intellectual disabilities (Scott & Havercamp, 2015), as well as potential compensatory mechanisms in caregiving, wherein milder depressive symptoms are buffered by caregivers being in charge of maintaining sleep, nutrition, hygiene, and daily activities. In future studies investigating the psychometric properties of the PAC-R, strategies to ensure consistency and reliability in clinical diagnoses may be helpful.

While the results of the current study indicate cause for optimism regarding the PAC-R, studies of both the PAC and the PAC-R to date have been almost exclusively based on the Norwegian-language version and conducted in a limited number of samples. Thus, further studies are needed in other samples and other languages, both with regard to the psychometric properties of the PAC-R, as well as its cut-off values, which may need to be adapted for other populations.

### Strengths and Limitations

As it was developed for autistic people with intellectual disabilities, a group which often struggles in self-reporting whether they have co-occurring symptoms of mental health disorders (Halvorsen, Kildahl, Kaiser et al., 2025; Helverschou et al., 2011), the PAC-R is based on informant reports. This is an inherent limitation when it comes to obtaining accurate information about mental health symptoms, which are subjective and experiential phenomena (Kildahl et al., 2024; Scott & Havercamp, 2018). Moreover, the current samples were obtained in the specific context of the Norwegian specialist health care system, and these findings may not be generalisable to community samples, as both samples were obtained from the specialist health services. Even though the samples were large for the populations in question, they were of limited size and involved a risk of potentially being statistically underpowered. The teams conducting the assessments in Sample 1 were not blind to the PAC scores of the participants and could have based their diagnoses partly on these scores, constituting a limitation for this sample. Moreover, few participants were diagnosed with serious mental health disorders in Sample 2, which may have affected the results. ADHD appears to be the most common co-occurring condition in autistic individuals (Lai et al., 2019), but as the PAC-R was not developed to identify co-occurring neurodevelopmental or behavioural disorders, ADHD and ODD were disregarded as diagnoses in the current study, constituting another limitation. On the other hand, the current study was able to verify the model fit of the PAC-R in two independent samples, as well as unearthing similar properties of the PAC-R as a screening tool, regardless of whether the diagnostician was blind to its results. These strengths of the current study highlight the continued cause for optimism regarding the PAC-R.

## Conclusion

This is the first study examining the screening properties of the PAC-R, which is based on the empirical development of the original PAC and its underlying factor structure. The PAC-R largely appears to have retained or improved on the properties of the original PAC as a screening tool. In addition, the PAC-R is more psychometrically sound than the original, with the subscale structure reflecting its underlying factor structure, and a previous study having shown its applicability for use in the evaluation of treatment. Even though further studies are needed to evaluate its psychometric properties, the PAC-R remains a promising mental health screening tool for autistic individuals.

## Supplemental Material

sj-pdf-1-aut-10.1177_13623613261480174 – Supplemental material for The Psychopathology in Autism Checklist–Revised (PAC-R): Development and Validation Supplemental material, sj-pdf-1-aut-10.1177_13623613261480174 for The Psychopathology in Autism Checklist–Revised (PAC-R): Development and Validation by Arvid Nikolai Kildahl, Sissel Berge Helverschou, Børge Mathiassen, Einar Heiervang and Marianne Berg Halvorsen in Autism

sj-pdf-2-aut-10.1177_13623613261480174 – Supplemental material for The Psychopathology in Autism Checklist–Revised (PAC-R): Development and Validation Supplemental material, sj-pdf-2-aut-10.1177_13623613261480174 for The Psychopathology in Autism Checklist–Revised (PAC-R): Development and Validation by Arvid Nikolai Kildahl, Sissel Berge Helverschou, Børge Mathiassen, Einar Heiervang and Marianne Berg Halvorsen in Autism

sj-pdf-3-aut-10.1177_13623613261480174 – Supplemental material for The Psychopathology in Autism Checklist–Revised (PAC-R): Development and Validation Supplemental material, sj-pdf-3-aut-10.1177_13623613261480174 for The Psychopathology in Autism Checklist–Revised (PAC-R): Development and Validation by Arvid Nikolai Kildahl, Sissel Berge Helverschou, Børge Mathiassen, Einar Heiervang and Marianne Berg Halvorsen in Autism

sj-pdf-4-aut-10.1177_13623613261480174 – Supplemental material for The Psychopathology in Autism Checklist–Revised (PAC-R): Development and Validation Supplemental material, sj-pdf-4-aut-10.1177_13623613261480174 for The Psychopathology in Autism Checklist–Revised (PAC-R): Development and Validation by Arvid Nikolai Kildahl, Sissel Berge Helverschou, Børge Mathiassen, Einar Heiervang and Marianne Berg Halvorsen in Autism

sj-pdf-5-aut-10.1177_13623613261480174 – Supplemental material for The Psychopathology in Autism Checklist–Revised (PAC-R): Development and Validation Supplemental material, sj-pdf-5-aut-10.1177_13623613261480174 for The Psychopathology in Autism Checklist–Revised (PAC-R): Development and Validation by Arvid Nikolai Kildahl, Sissel Berge Helverschou, Børge Mathiassen, Einar Heiervang and Marianne Berg Halvorsen in Autism

## Data Availability

The ethical approvals do not allow for data sharing, and the data for the current study are not available due to their sensitive nature.
