# Supplementary material for: The Psychopathology in Autism Checklist–Revised (PAC-R): Development and Validation
Source: Autism. 2026 Aug 30;30(11):2794–804. doi: 10.1177/13623613261480174 (PMC13687617; doi:10.1177/13623613261480174)
Supplement: Supplementary file 1 [file sj-pdf-1-aut-10.1177_13623613261480174.pdf]

PAC/PAC-R (Psychopathology in Autism Checklist, Psychopathology in Autism Checklist-Revised)

Screening checklist for identifying symptoms of mental health problems in autistic people

Background information (Tick the appropriate box)

<table>
<tr><td>Client code</td><td colspan="4"></td></tr>
<tr><td>Age of client</td><td>Gender:</td><td colspan="2">1 Male</td><td>2 Female</td></tr>
<tr><td>Informant:</td><td colspan="3">1 Contact person (home/institution)</td><td>2 Close relative</td></tr>
<tr><td>Living arrangements:</td><td colspan="3">1 Local authority housing</td><td>2 With parents</td></tr>
<tr><td>How long have you known the client:</td><td colspan="2">Years</td><td colspan="2">Months</td></tr>
<tr><td>Does the client take regular medication?</td><td colspan="2">1 Yes</td><td colspan="2">2 No</td></tr>
<tr><td colspan="5">If the answer to the question above is Yes, what medication is taken?
Do not give the dose.</td></tr>
<tr><td>How does the client communicate?</td><td colspan="2">1. Speaks using whole phrases/sentences</td><td colspan="2">2. Uses speech plus alternative communication (e.g. Makaton, symbols, computer)</td></tr>
<tr><td></td><td colspan="2">3.Uses a few words, signs and gestures</td><td colspan="2">4. Uses own made-up signs/gestures</td></tr>
<tr><td colspan="5">Has the client’s life situation changed significantly during the last year?
1 Yes
If the answer is Yes, what changes have there been? (key words)

2 No</td></tr>
<tr><td>Date of completion of form</td><td colspan="4">------------- / ---------- ----------</td></tr>
</table>

© 2010 The National Autism Unit, Oslo University Hospital, Norway.
© 2026 Regional Section Mental Health, Intellectual Disabilities/Autism, Oslo University Hospital, Norway.

For each item, state to what extent the following are a problem for the client as he/she is at present (left-hand column). Indicate any change in the symptom/ behaviour description compared to usual behaviour (right-hand column).

		**Extent of problems**				**Change from usual behaviour**		
		Not a problem	Mild	Moder ate	Severe	Wors-ened	Un-changed	Im-proved
1	Is passive during extended periods of the day							
2	Takes little initiative in performing daily tasks							
3	Appears agitated							
4	Has difficulty sleeping							
5	Withdraws from or avoids social situations							
6	Reacts with irritation to trivial occurrences							
7	Self-injures							
8	Loses the thread or track of the conversation or activity							
9	Destroys surroundings							
10	Is abnormally anxious to get confirmation, for example about some specific event that is to take place, etc.							
11	Violent towards other people							
12	Appears to be tired during the day							
13	Sees things that other people do not see/ visual hallucinations							
14	Is excessively concerned as to whether a task has been carried out “correctly” for him/her							
15	Shakes/trembles							
16	Complains about pains, for example in the stomach or chest							
17	Gets disoriented in familiar situations or in familiar places							
18	Hyper-ventilates, rapid and shallow breathing							
19	Checks repeatedly if doors are locked, switches are off or on etc.							
20	Needs a great deal of help to get going in the morning							
21	Unusual and fixed beliefs that are impossible to change / delusions							
22	Has difficulties stopping obsessive or ritualised behaviours							
23	Acts in a threatening manner: verbally, facial expression, body language etc.							
24	Shows loss of energy							
25	Sweats, feels clammy							
26	Has sudden changes of mood							
27	Has a special behaviour pattern; follows fixed routines, touches certain objects etc.							
28	Shows fear of specific situations							
29	Behaves in a way that indicates listening for something that is non-existent, auditory hallucinations							
30	Has gained / lost weight							
31	Has enlarged pupils							
32	Appears nervous							
33	Repeatedly washes things – clothes, furniture etc.							
34	Is disoriented / confused in interactions with others							
35	Shows little interest in things he/she previously enjoyed /was interested in							
36	Excessive washing/brushing teeth etc.							
37	Is unable to follow familiar instructions							
38	Has a high level of motor activity /overactive							
39	Appears sad and dejected							
40	Needs to carry out something a fixed number of times							
41	Has a high level of communicative activity/ flood of words							
42	Spends more time on compulsive or ritualised behaviour							

© 2010 The National Autism Unit, Oslo University Hospital, Norway.
© 2026 Regional Section Mental Health, Intellectual Disabilities/Autism, Oslo University Hospital, Norway.
