# Supplementary material for: The Psychopathology in Autism Checklist–Revised (PAC-R): Development and Validation
Source: Autism. 2026 Aug 30;30(11):2794–804. doi: 10.1177/13623613261480174 (PMC13687617; doi:10.1177/13623613261480174)
Supplement: Supplementary file 2 [file sj-pdf-2-aut-10.1177_13623613261480174.pdf]

Oslo universitetssykehus

Psychopathology in Autism Checklist-Revised (PAC-R) – scoring

Any MH disorder, 32 items– cut-off ≥1.8

ITEM	Item label	Score
1	Is passive	
2	Takes little initiative	
3	Appears agitated	
4	Has difficulty sleeping	
5	Withdraws from…	
8	Loses the tread or track	
10	Is abnormally anxious…	
12	Appears to be tired	
13	Sees things that others…	
14	Is excessively concerned	
15	Shakes/trembles	
16	Complains about pain	
17	Gets disoriented in familiar	
18	Hyperventilates	
19	Checks repeatedly	
20	Needs a great deal of help	
22	Has difficulties interrupting	
24	Shows loss of energy	
25	Sweats, feels clammy	
27	Has a special behaviour	
28	Shows fear of specific	
29	Auditory hallucinations	
30	Has gained/lost weight	
32	Appears nervous	
34	Is disoriented/confused	
35	Shows little interest in…	
37	Is unable to follow familiar	
38	Has a high level of motor…	
39	Appears sad and dejected	
40	Needs to carry out	
41	Has a high level of comm…	
42	Spends more time	
**Total score**		
**Mean score**		

Aggression/self-injury, 6 items – no cut-off

ITEM	Item label	Score
6	Reacts with irritation	
7	Self-injures	
9	Destroys surroundings	
11	Violent towards others	
23	Acts in a threatening manner	
26	Sudden changes of mood	
**Total score**		
**Mean score**		

Summary of mean scores

Any mental health disorder (≥1.8)	
Psychosis (≥1.8)	
Depression (≥2.2)	
Anxiety (≥2.1)	
OCD (≥2.3)	

Psychosis, 6 items – cut-off ≥1.8

ITEM	Item label	Score
8	Loses the tread or track	
13	Sees things that others…	
17	Gets disoriented in familiar	
29	Auditory hallucinations	
34	Is disoriented/confused	
37	Is unable to follow familiar	
**Total score**		
**Mean score**		

Depression, 8 items – cut-off ≥2.2

ITEM	Item label	Score
1	Is passive	
2	Takes little initiative	
12	Appears to be tired	
20	Needs a great deal of help	
24	Shows loss of energy	
30	Has gained/lost weight	
35	Shows little interest in…	
39	Appears sad and dejected	
**Total score**		
**Mean score**		

Anxiety, 9 items – cut-off ≥2.1

ITEM	Item label	Score
3	Appears agitated	
10	Is abnormally anxious	
15	Shakes/trembles	
18	Hyperventilates	
25	Sweats, feels clammy	
28	Shows fear of specific	
32	Appears nervous	
38	Has a high level of motor…	
41	Has a high level of comm..	
**Total score**		
**Mean score**		

OCD, 6 items – cut-off ≥2.3

ITEM	Item label	Score
14	Is excessively concerned	
19	Checks repeatedly	
22	Has difficulties stopping	
27	Has a special behaviour	
40	Needs to carry out	
42	Spends more time	
**Total score**		
**Mean score**		

Indication for further assessment?

Any subscales over the cut-off?
Any other indications of mental health disorder?

YES	
NO	

© 2026 Regional section mental health, intellectual disabilities/autism, Oslo University Hospital. Developed in collaboration with NevSom national centre of expertise.
