# Supplementary material for: The Psychopathology in Autism Checklist–Revised (PAC-R): Development and Validation
Source: Autism. 2026 Aug 30;30(11):2794–804. doi: 10.1177/13623613261480174 (PMC13687617; doi:10.1177/13623613261480174)
Supplement: Supplementary file 3 [file sj-pdf-3-aut-10.1177_13623613261480174.pdf]

Oslo universitetssykehus

Psychopathology in Autism Checklist-Revised (PAC-R)
Screening checklist for mental health symptoms in autistic individuals

Introduction for health professionals

The Psychopathology in Autism Checklist-Revised (PAC-R; Kildahl et al., 2026b) is a screening checklist, completed by caregivers, originally designed to identify autistic individuals (adults) with intellectual disabilities in need of more comprehensive mental health assessment. The PAC-R is not a diagnostic instrument but a tool to aid the diagnostic process. Individuals with high scores on the PAC-R should be referred for a more comprehensive psychiatric examination.

The PAC-R should be scored by informants with detailed knowledge of the individual, such as primary carers or family members. However, the interpretation of the ratings should be done by professionals in specialised health services who are familiar with the co-occurrence of autism, intellectual disabilities and mental health disorders. Diagnosing mental health disorders in this group is complex. An accurate diagnostic assessment depends on distinguishing between autism characteristics, consequences of difficulties in intellectual functioning and symptoms of a co-occurring mental health disorder. This requires a comprehensive patient history, including identification of changes in the individual's functioning over time. This includes potential changes to autism characteristics, other behaviours, well-being, uneasiness and distress, conventional mental health symptoms, as well as the recognition of atypical or idiosyncratic symptoms. To obtain this information, collaboration with family members and others who know the person well is essential.

The original PAC (Helverschou et al., 2008, 2009) was developed in collaboration between the Psychiatric Department for Adults with Intellectual Disability and Autism and the Autism Unit, Oslo University Hospital. The revision, PAC-R was developed in collaboration between the same institutions, though under other department names.

Description

The PAC-R comprises 42 items, of which 38 are used in scoring. These 38 items are distributed across 5 subscales: *psychosis* (6 items), *depression* (8 items), *anxiety* (9 items), *obsessive-compulsive disorder* (OCD; 6 items) and *aggression/self-injurious behaviour* (6 items). In addition, an index is scored for "any mental health disorder" (32 items). This index includes all the items from the four disorder-specific subscales (psychosis, depression, anxiety, OCD), and three other items (social withdrawal, sleep difficulties, pain).

In the first validation study (Helverschou et al., 2009), the original PAC was found to distinguish between autistic adults with intellectual disabilities with and without co-occurring mental health disorders, and to a certain extent between individuals with different mental health disorders. Later studies made similar findings (Helverschou et al., 2021), and these psychometric properties have been found to be retained for the PAC-R (Kildahl et al., 2026b). In addition, the internal consistency of the PAC-R has been found to be good to acceptable (Kildahl et al., 2026a, 2026b). Finally, the subscales of the PAC-R have been found to show longitudinal measurement invariance, indicating that they are appropriate for use in evaluation of mental health treatment intervention in autistic adults with intellectual disabilities (Kildahl et al., 2026a). Other findings indicate that the PAC/PAC-R shows structural validity also among autistic children and adolescents (Halvorsen et al., 2025).

© 2010 The National Autism Unit, Oslo University Hospital, Norway.
© 2026 Regional Section Mental Health, Intellectual Disabilities/Autism, Oslo University Hospital, Norway.

Oslo universitetssykehus

Using the Rating Scale

Items are presented in a random order. The items describe a broad range of potential problems and symptoms. Not all items will be relevant to all individuals, but it is important that the informants rate all the items in order to be able to calculate the subscale scores. Each item is assessed in two domains: "Extent of problems" addresses the current extent of problems (1 = no problem, 2 = minor problem, 3= moderate problem, 4 = severe problem). "Change from usual behaviour" addresses whether the behaviour has changed in relation to the specific individual's previous or typical patterns of behaviour (Worsened, Unchanged, Improved).

Some of the items describe phenomena that are not directly observable. Even if it is difficult, all items should be scored by the informant to the best of their ability. Such considerations are particularly difficult in relation to individuals with limited verbal language skills. However, clinical experience indicates that people with thorough knowledge of the specific individual are able to make such interpretations.

Interpreting the ratings

The item scores are used to calculate the mean score for each subscale and the "any mental health disorder" index. Missing items are managed subscale-wise, by replacing the missing item score by the mean score for the remaining items on the same subscale. The maximum number of substitutions are 2 items for the anxiety and depression subscales, and 1 item for the psychosis, OCD, and aggression/self-injurious behaviour subscales.

Cut-off values were established in the PAC-R validation study (Kildahl et al., 2026b): Psychosis 6 items - cut-off ≥1.8; Depression 8 items - cut-off ≥2.2; Anxiety 9 items - cut-off ≥2.1; OCD 6 items - cut-off ≥2.3. There is no cut-off for the aggression/self-injurious behaviour subscale. In addition, the "any mental health disorder" index has 32 items and a cut-off of ≥1.8. Findings from the validation study indicate that there may be a risk of false negatives on the "any mental health disorder" index, and a risk of false positives on the four disorder-specific subscales, and this should be considered when interpreting the scores. Notably, there may be other indications of mental health disorder not captured by the PAC-R that may warrant a comprehensive assessment despite low scores. Thus, subscale scores should be interpreted in context of other available information about the individual.

References:

Halvorsen, M. B., Kildahl, A. N., Thimm, J. C., & Helverschou, S. B. (2025). Structural validity of the Psychopathology in Autism Checklist among children and adolescents. *Journal of Intellectual Disability Research, 68*(10), 1123-1131. https://doi.org/10.1111/jir.70012

Helverschou, S. B., Bakken, T. L., & Martinsen, H. (2008). Identifying symptoms of psychiatric disorders in people with autism and intellectual disability: An empirical conceptual analysis. Mental Health Aspects of Developmental Disabilities, 11(4), 105 – 115.

Helverschou, S. B., Bakken, T. L., & Martinsen, H. (2009). The Psychopathology in Autism Checklist (PAC): a pilot study. *Research in Autism Spectrum Disorders, 3*, 179 – 195. https://doi.org/10.1016/j.rasd.2008.05.004

Helverschou, S. B., Ludvigsen, L. B., Hove, O., & Kildahl, A.N. (2021). Psychometric properties of the Psychopathology in Autism Checklist (PAC). *International Journal of Developmental Disabilities, 67*(5), 318-326. https://doi.org/10.1080/20473869.2021.1910779

Kildahl, A. N., Halvorsen, M. B., Hellerud, J. M. A., & Helverschou, S. B. (2026a). Factor structure and longitudinal measurement invariance of the Psychopathology in Autism Checklist. *Journal of Autism and Developmental Disorders.* https://doi.org/10.1007/s10803-026-07363-8

Kildahl, A. N., Helverschou, S. B., Mathiassen, B. I., Heiervang, E., & Halvorsen, M. B. (2026b). The Psychopathology in Autism Checklist–Revised (PAC-R): Development and validation. *Autism.* https://doi.org/10.1177/13623613261480174

Contact information

Arvid Nikolai Kildahl – uxarvk@ous-hf.no

© 2010 The National Autism Unit, Oslo University Hospital, Norway.
© 2026 Regional Section Mental Health, Intellectual Disabilities/Autism, Oslo University Hospital, Norway.
