# Supplementary material for: The Psychopathology in Autism Checklist–Revised (PAC-R): Development and Validation
Source: Autism. 2026 Aug 30;30(11):2794–804. doi: 10.1177/13623613261480174 (PMC13687617; doi:10.1177/13623613261480174)
Supplement: Supplementary file 4 [file sj-pdf-4-aut-10.1177_13623613261480174.pdf]

Oslo universitetssykehus

Psychopathology in Autism Checklist (PAC)
Screening checklist for identifying psychiatric symptoms in individuals with autism

Rating sheet

GAP 12 items – cut-off ≥2.0			Psychosis 10 items - cut-off ≥2.3		
**ITEM**	**Item label**	**Score**	**ITEM**	**Item label**	**Score**
1 - Gen1	Is passive				
2 – Gen2	Takes little initiative				
3 - Gen3	Appears agitated		8 – Psy1	Loses the tread or track….	
4 - Gen4	Has difficulty sleeping		13 - Psy2	Sees things that other s ….	
5 – Gen5	Withdraws from ….		17 – Psy3	Gets disoriented in familiar..	
6 - Gen6	Reacts with irritation		21 – Psy4	Delusions	
7 – Gen7	Self-injures		23 – Psy5	Acts in a threatening manner	
9 - Gen8	Destroys surroundings		26 - Psy6	Has sudden changes of mood	
10 – Gen9	Is abnormally anxious..		29 – Psy7	Auditory hallucinations	
11 - Gen10	Violent toward others		34 – Psy8	Is disoriented /confused…	
16 – Gen11	Complains about pain ..		38 – Psy9	Has a high level of motor …	
42 – Gen12	Spends more time …		41 - Psy10	Has a high level of comm….	
**Total score**			**Total score**		
**Mean score**			**Mean score**		

OCD 7 items - cut-off ≥2.4			Depression 7 items - cut-off ≥2.0		
14 – OCD 1	Is excessively concerned		12 - Dep1	Appears to be tired	
19 – OCD 2	Checks repeatedly		20 – Dep2	Needs a great deal of help	
22 - OCD3	Has difficulties stopping		24 - Dep3	Shows loss of energy	
27 - OCD4	Has a special behaviour		30 – Dep4	Has gained / lost weight	
33 – OCD5	Repeatedly washes...		35 - Dep5	Shows little interest in..	
36 – OCD6	Excessive washing		37 – Dep6	Is unable to follow familiar..	
40 - OCD7	Needs to carry out ..		39 - Dep7	Appears sad and dejected	
**Total score**			**Total score**		
**Mean score**			**Mean score**		

Anxiety 6 items - cut-off ≥1.8			Summary of mean scores	
15 – Anx1	Shakes / trembles			
18 – Anx2	Hyper-ventilates			
25 – Anx3	Sweats, feel clammy			
28 – Anx4	Shows fear of specific		**GAP (≥2.0)**	
31 – Anx5	Has enlarged pupils		**Psychosis (≥2.3)**	
32 - Anx6	Appears nervous		**OCD (≥2.4)**	
**Total score**			**Depression (≥2.0)**	
**Mean score**			**Anxiety (≥1.8)**	

Is the GAP mean score above cut-off? ☐ **YES** ☐ **NO**

Is the mean score above cut-off on any of the other subscales? If, yes, which one

……………………………………………………

Is there reason to suspect that the individual suffers from a psychiatric disorder?

☐ **YES** ☐ **NO**

© 2010 The National Autism Unit, Oslo University Hospital, Norway.
Developed in cooperation with Psychiatric Department for Intellectual Disability /Autism.
