# Supplementary material for: The Psychopathology in Autism Checklist–Revised (PAC-R): Development and Validation
Source: Autism. 2026 Aug 30;30(11):2794–804. doi: 10.1177/13623613261480174 (PMC13687617; doi:10.1177/13623613261480174)
Supplement: Supplementary file 5 [file sj-pdf-5-aut-10.1177_13623613261480174.pdf]

Psychopathology in Autism Checklist (PAC)
Screening checklist for psychiatric symptoms in adults with autism

Introduction for health professionals

The Psychopathology in Autism Checklist (PAC) is a screening checklist, completed by caregivers, designed to identify adults with autism and ID in need of psychiatric services. The PAC is not a diagnostic instrument but a tool to aid the diagnostic process. Individuals who obtain high scores on the PAC should be referred for a comprehensive psychiatric examination.
The PAC is rated by informants with detailed knowledge of the individual, such as primary carers or family members. However, the interpretation of the ratings should be performed by professionals in specialised health services who are familiar with autism, ID and psychiatric disorders.
The PAC was developed in collaboration between the Psychiatric Department for Adults with Intellectual Disability and Autism and the Autism Unit, Oslo University Hospital.

Diagnosing psychiatric disorders in this group is complex. An accurate diagnostic assessment depends on distinguishing between symptoms that represent the autistic condition and symptoms that indicate additional psychiatric conditions. Thus, the diagnostics should be based on identification of qualitative "changes" in the individual's long-standing features of autism, the presence of conventional psychiatric symptoms, and the recognition of idiosyncratic or atypical symptoms. It is important to co-operate with the family and others who know the person well, in order to obtain information on well-being or uneasiness, changes in premorbid or typical patterns of behavior and mood, and to provide information on idiosyncratic or atypical symptoms.

Description

The PAC comprises 42 items distributed across 5 subscales; *psychosis* (10 items), *depression*, (7 items), *anxiety disorders* (6 items), *obsessive-compulsive disorder* (OCD) (7 items) and *general adjustment problems* (12 items). Thirty items represent symptoms assessed as specific to one of four major psychiatric disorders and not to autism. Twelve items are assessed as indicators of general problems often observed in individuals with adjustment problems and/or psychiatric disorders; e.g. sleep disturbances, self-harm, irritability, passivity, and restlessness.

In the first validation study (Helverschou, Bakken & Martinsen, 2009), the PAC was found to discriminate between adults with autism and ID with and without psychiatric disorders, and to a certain extent between individuals with different psychiatric disorders, especially psychosis and OCD. The psychometric properties, i.e. internal consistency computed by Cronbach's α and inter-rater agreement computed by Cohen's Kappa were also acceptable.

Using the Rating Scale

Items are presented in random order. It is important that the informants are blind to which subscale each of the items belongs. The items describe a broad spectre of possible problems and symptoms. Not all items will be relevant to all individuals, but it is important that the informants rate all the items. Each item is assessed on 2 domains: "Extent of problems" - addresses the current extent of problems (1 = no problem; 2= minor problem; 3= moderate problem; 4 = severe problem). "Change from usual behavior" - addresses whether the

© 2010 The National Autism Unit, Oslo University Hospital, Norway.
Developed in cooperation with Psychiatric Department for Intellectual Disability /Autism.

behavior has changed in relation to premorbid or typical patterns of behavior (Worsened, Unchanged, and Improved).

Some of the items describe phenomena that are not directly observable. Even if it is difficult, it is desirable that the informant attempts to give an opinion on whether or not the item is relevant to the target individual. Such considerations are particularly difficult in relation to individuals without spoken language. However, clinical experience indicates that people with thorough knowledge of the target individual are able to make such interpretations correctly.

Interpreting the ratings

The interpretation of the ratings should be performed by professionals in specialised health services familiar with both autism and ID, and psychiatric disorders.

Procedure: The values of the rating scale range from 1 to 4 (1 = no problem; 2= minor problem; 3= moderate problem; 4 = severe problem). Cut-off values for psychiatric disorders and severe general adjustment problems have been established based on the results of the PAC validation study. General Adjustment problems (GAP) 12 items - cut-off ≥2.0 = severe adjustment problems; Psychosis 10 items - cut-off ≥2.3; Depression 7 items - cut-off ≥2.0; Anxiety disorders 6 items - cut-off ≥1.8; OCD 7 items - cut-off ≥2.4

A two-step procedure secures identification of individuals with a possible psychiatric disorder. First, individuals with *severe general adjustment problems* are identified, i.e. average GAP score above cut-off. Thereafter, those individuals who obtain an average score above cut-off for any of the psychiatric subscales are classified as suspected as having a psychiatric disorder. They should be referred to a comprehensive psychiatric examination.

Publications:

Bakken, T.L., Friis, S., Lovoll, S., Smeby, N.A., Martinsen, H. (2007). Behavioral Disorganisation as an Indicator of Psychosis in Adults with Intellectual Disability and Autism. Mental Health Aspects of Developmental Disabilities, 10:2, 37-46.

Bakken, T. L., Helverschou, S. B., Eilertsen, D. E., Hegglund, T., Myrbakk, E., & Martinsen, H. (2010). Psychiatric disorders in adolescents and adults with autism and intellectual disability: A representative study in one county in Norway. Research in Developmental Disabilities, 31, 1669-1677.

Bakken, T.L. and Høidal, S.H. (2014). Asperger Syndrome or schizophrenia, or both? Case identification of 12 adults in a specialised psychiatric inpatient unit. Int J Developemental Disabilities, vol.60, No 4, 215 – 225.

Helverschou, S.B., Bakken, T.L. & Martinsen, H. (2008). Identifying symptoms of psychiatric disorders in people with autism and intellectual disability: An empirical conceptual analysis. Mental Health Aspects of Developmental Disabilities, Vol. 11 – No. 4, 105 – 115.

Helverschou, S.B., Bakken, T.L., & Martinsen, H. (2009). The Psychopathology in Autism Checklist (PAC): a pilot study. Research in Autism Spectrum Disorders, 3, 179 – 195.

Helverschou, S.B. & Martinsen, H. (2011). Anxiety in people diagnosed with autism and intellectual disability: Recognition and phenomenology. Research in Autism Spectrum Disorders, 5, 377-387.

Helverschou, S.B., Kildahl, A.N. & Bakken, T.L. (2020). Checklists And Structured Interviews, In: Matson, J. (ed): Handbook of dual diagnoses. Assessment and Treatment in Persons with Intellectual Disorders, pp.167-193. Springer Nature Switzerland AG. ISBN 978-3-030-46834-7.

Helverschou, S.B., Bakken, T.L., Berge, H., Bjørgen, T.G., Botheim, H., Hellerud, J.A., Helseth, I., Hove, O., Johansen, P.A., Kildahl, A.N., Ludvigsen, L.B., Nygaard, S., Rysstad, A., Wigaard, E., & Howlin, P. (2020). Preliminary findings from a nationwide, multi-centre mental health service for individuals with autism spectrum disorders and intellectual disability. Journal of Policy and Practice in Intellectual Disabilities DOI: 10.1111/jppi.12366

Halvorsen, M. & Helverschou, S.B. (2020). Identifisering av psykiske helsevansker hos personer med autismespekterforstyrrelser og psykisk utviklingshemming. Tidsskrift for Norsk Psykologforening; Volum 3.

Helverschou, S.B., Ludvigsen, L.B., Hove, O., Arvid Nikolai Kildahl, A.N. (2021). Psychometric properties of the Psychopathology in Autism Checklist (PAC). International Journal of Developmental Disabilities. https://doi.org/10.1080/20473869.2021.1910779

Contact information

Sissel Berge Helverschou: shelvers@ous-hf.no and Trine Lise Bakken: UXTLBA@ous-hf.no

© 2010 The National Autism Unit, Oslo University Hospital, Norway.
Developed in cooperation with Psychiatric Department for Intellectual Disability /Autism.
